# Cryopreserved placental biopsies maintain mitochondrial activity for high-resolution respirometry

**DOI:** 10.1186/s10020-023-00645-2

**Published:** 2023-04-03

**Authors:** Matteo Giovarelli, Anais Serati, Silvia Zecchini, Fabiola Guelfi, Emilio Clementi, Chiara Mandò

**Affiliations:** 1 Department of Biomedical and Clinical Sciences, Università Degli Studi Di Milano, Via G.B. Grassi 74, 20157 Milan, Italy; 2 Department of Woman Mother and Neonate ‘V. Buzzi’ Children Hospital, ASST Fatebenefratelli Sacco, 20154 Milan, Italy; 3 Scientific Institute, IRCCS Eugenio Medea, Via Don Luigi Monza 20, 23842 Bosisio Parini, Italy

**Keywords:** Placental biopsies, Mitochondria, High-resolution respirometry, Cryopreservation

## Abstract

**Background:**

High-resolution respirometry (HRR) of human biopsies can provide useful metabolic, diagnostic, and mechanistic insights for clinical research and comparative medical studies. Fresh tissues analysis offers the potential best condition, the drawback being the need to use them shortly after dissection for mitochondrial respiratory experiments. The development of effective long-term storage protocols for biopsies that allow the assessment of key Electron Transport System (ETS) parameters at later stages is thus a major need.

**Methods:**

We optimised a cryopreservation protocol that preserves mitochondrial membranes intactness, otherwise affected by direct tissue freezing. The protocol is based on a gradual freezing step from on-ice to liquid nitrogen and − 80 °C storage using a specific DMSO-based buffer.

**Results:**

Placenta is a suitable tissue to design and test the effectiveness of long-term storage protocols being metabolically active foetal tissue with mitochondrial dysfunctions contributing to placental disease and gestational disorders. Here we designed and tested the effectiveness of the cryopreservation protocol using human placenta biopsies; we measured the ETS activity by HRR of placenta specimens comparing fresh, cryopreserved, and snap frozen conditions.

**Conclusions:**

By this protocol, Oxygen Consumption Rate (OCR) measurements of fresh and cryopreserved placental specimens are comparable whereas snap frozen procedure impairs mitochondrial activity.

**Graphical Abstract:**

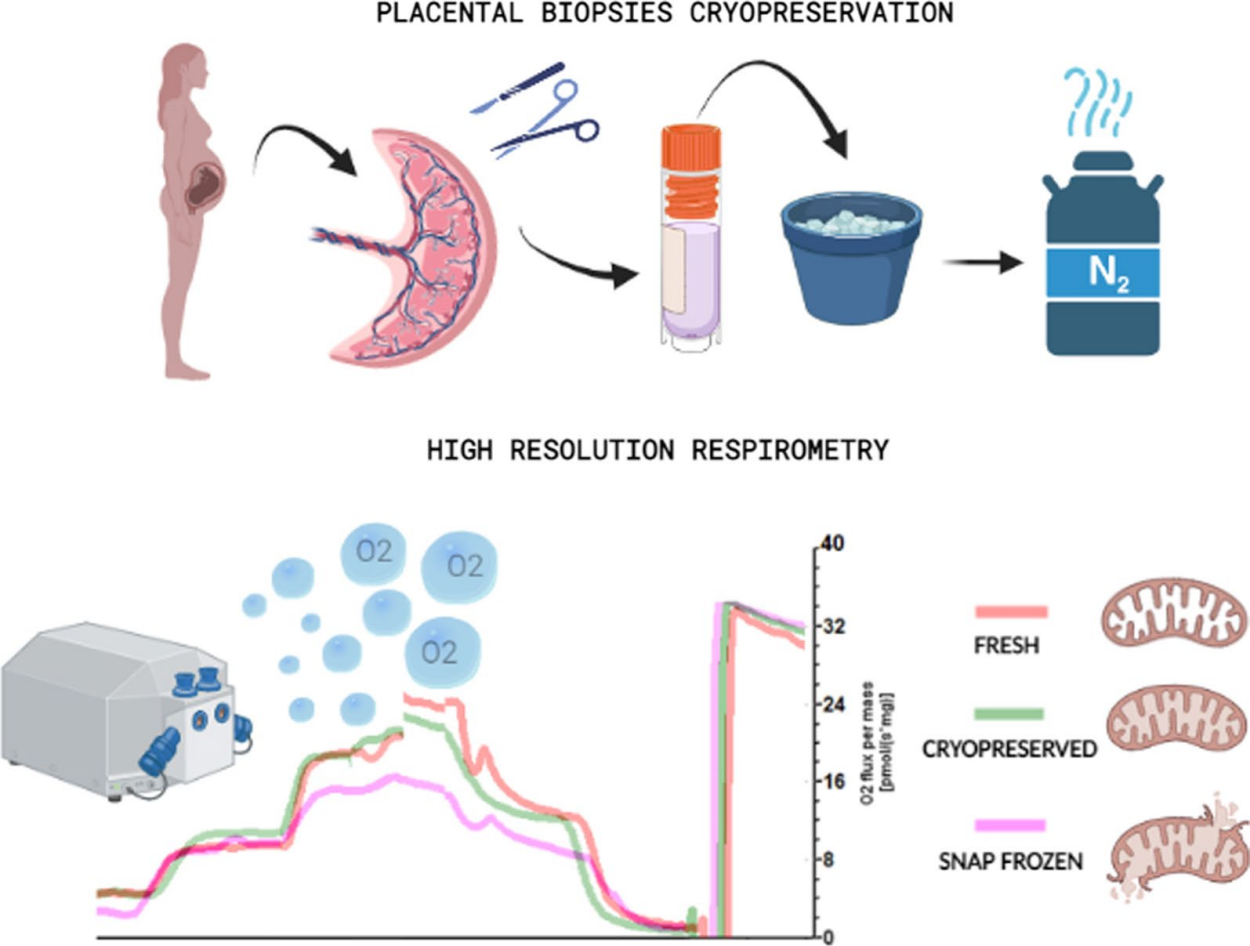

## Introduction

Oxygen consumption analysis provides information on the activity of the mitochondrial Electron Transfer System (ETS) and the coupling between the oxidation of metabolic substrates and the phosphorylation of ADP to ATP (OXPHOS). This is useful in studies aimed at understanding the role of mitochondria as the main hub for cellular homeostasis (Dan Dunn et al. 2015; Spinelli and Haigis 2018). High Resolution Respirometry (HRR) is the gold standard for ETS measurements; it allows a deep understanding of metabolic changes in specific physiological states and disease conditions (Nolfi-Donegan et al. 2020; Awadhpersad and Jackson 2021; Roden 2022) and is now used extensively on tissue biopsies (Hütter et al. 2006). Although the use of fresh tissue is the ideal condition, it is not always possible to process immediately patients’ specimens for HRR; therefore, a reliable long-term freezing storage for human biopsies is a key requirement for implementing clinical research also in a retrospective type of analyses.

Bioenergetic studies of mitochondrial activity done by snap freezing provide unreliable results since it damages mitochondrial membranes triggering the loss of cytochrome c (cyt c), the dissipation of mitochondrial membrane potential, and eventually hampering ETS activity (Larsen et al. 2012). Only few studies have addressed comprehensively the intactness of mitochondrial function after biopsies cryopreservation methods; they show variable degrees of effectiveness and conflicting results (Colleoni et al. 2012; Fisher et al. 2019; Acin‐Perez et al. 2020). Besides, the use of cellular homogenates and isolated mitochondria for HRR has intrinsic limitations since the absence of the physiological cellular milieu for mitochondria may affect results interpretation (Acin‐Perez et al. 2020; Zuccolotto-dos-Reis et al. 2021). In addition, mitochondrial isolation methods can lead to the loss of damaged organelles, hence possibly operating a mitochondrial population selection (Picard et al. 2011). Therefore, the setting of standardised protocols for long-term biopsies storage and mitochondrial analysis is a major priority (Kuznetsov et al. 2003; Colleoni et al. 2012; Larsen et al. 2012; García-roche et al. 2018).

The placenta is a high energy-demanding and metabolically active tissue playing a central role in growth and development of the foetus by facilitating oxygen and nutrient supply, and waste removal (Sferruzzi-Perri and Camm 2016). Mammals’ placenta mainly relies on OXPHOS ATP supply thus involving a significant oxygen requirement (50–70% of oxygen from uterine circulation) and placental mitochondria need to metabolically adapt throughout gestation (Schneider 2000; Holland et al. 2017). For this reason, mitochondrial dysfunctions are involved in placental pathophysiology leading to gestational disorders (Hastie and Lappas 2014; Mele et al. 2014; Anelli et al. 2018; Mandò et al. 2018; Cetin et al. 2020; Diceglie et al. 2021). Therefore, Oxygen Consumption Rate (OCR) analysis of placental biopsies provides a valuable tool to identify mitochondrial dysfunctions leading to energetic, metabolic, and oxidative stresses and associated with pregnancy disorders (Mandò et al. 2014; Holland et al. 2018). Although mitochondrial respiration on fresh placental biopsies has been established using standardised HRR protocols (Holland et al. 2017, 2018), to date placental specimens cryopreservation has not been effectively optimised and it is not used in delivery-associated sampling routine. In 2012, Murray and co-workers described a simple cryopreservation protocol for placental biopsies limited to complex I-dependent respiratory state; while simple, it showed a decrease of both state 2 and 3 respiration suggestive of increased mitochondrial uncoupling (Colleoni et al. 2012). Another cryopreservation protocol described by Perkins and co-workers, though not impairing ETC activity, was restricted to mitochondria preparations from placental cytotrophoblasts and syncytiotrophoblasts (Fisher et al. 2019).

Here we describe an optimised cryopreservation protocol for fresh placental biopsies developed from an already used protocol for bioptic animal liver and diaphragm muscle specimens (García-roche et al. 2018; Giovarelli et al. 2021). The protocol we developed yielded consistent results on human placental specimens and allowed the preservation of intact mitochondrial ETS activity.

## Materials and methods

### Placental biopsies collection

Pregnant women were enrolled at the Obstetric Unit of the Vittore Buzzi Children Hospital (ASST Fatebenefratelli-Sacco) in Milan. The study protocol was approved by the university hospital ethical committee (Prot. N. 17739/2018) and all participants gave written informed consent for personal data treatment and biological specimens’ collection. Five Caucasian pregnant women, with normal weight pregestational Body Mass Index (BMI) (18.5 kg/m^2^ ≤ BMI < 25 kg/m^2^), aged 18–42 years, with single spontaneous term physiological pregnancies were recruited at elective Caesarean Section (in absence of labor) (eCS). Mothers presenting any pathology or pregnancy complication were excluded. Placentas were collected immediately after eCS and sampled in a central site of the placental disc, between the cord insertion and the disc side. Placental chorionic villi biopsies of ~ 1 cm^3^ were collected from the maternal side of the placenta, after elimination of maternal decidua. Collected samples were carefully washed in PBS to eliminate excessive blood, then immediately transferred to cold BIOPS buffer (2.77 mMCaK2EGTA, 7.23 mM K2EGTA, 5.77 mM Na2ATP, 6.56 mM MgCl2·6H2O, 20 mM taurine, 15 mM Na2 phosphocreatine, 20 mM imidazole, 0.5 mM dithiothreitol, 50 mM MES, pH 7.1) and quickly transported (< 2 h, 1.5 h average,) to the laboratory in ice.

### Cryopreservation

Placental biopsies were cryopreserved as described previously (García-roche et al. 2018; Giovarelli et al. 2021). Placental biopsies in ice-cold BIOPS buffer were quickly dried in blotting paper and soaked in cryovials containing 1 mL of ice-cold modified University of Wisconsin solution (UW) (20 mM histidine, 20 mM succinate, 3 mM glutathione, 1 μM leupeptin, 2 mM glutamate, 2 mM malate, 2 mM ATP, 0.5 mM EGTA, 3 mM MgCl2·6H2O, 60 mM MOPS, 20 mM taurine, 10 mM KH2PO4, 20 mM HEPES, 110 mM sucrose, 1 g/L bovine serum albumin (BSA) and 10% (v/v) dimethyl sulfoxide (DMSO) (Sakata et al. 1997)). DMSO was added to the solution immediately before cryopreservation. The cryopreservation was through gradual freezing method allowing cellular components preservation. Once in cryovials, samples were left on ice for 8 minutes (min) (2–4 °C), then exposed to nitrogen vapours for 10 min hanged in a canister placed 10–15 cm below the neck tube level of the nitrogen tank which was kept with the lid closed and without the cap plug. Thereafter, the samples were submerged in liquid nitrogen for 10 min minimum and finally stored at − 80 °C. In order to compare direct freezing procedure, pieces sampled from the same placental site were snap frozen in parallel by immerging the cryovials directly in nitrogen for at least 10 min and then stored at − 80 °C. Cryopreserved and snap frozen samples were kept at least 2 months in − 80 °C before HRR, histological and western blot analyses. Right before the experiments, samples were thawed at room temperature and washed in a tube with 5 ml of ice-cold MIR06 medium (0.5 mM EGTA, 3 mM MgCl_2_, 60 mM K-lactobionate, 20 mM taurine, 10 mM KH_2_PO_4_, 20 mM Hepes, 110 mM sucrose and 1 g/l bovine serum albumin fatty acid-free, 280 U/ml catalase, pH 7.1); for HRR, the specimens were mechanically dissociated with a pair of forceps on a petri dish with chilled BIOPS to obtain ~ 5 mg pieces.

### Permeabilisation

Selective plasma membrane permeabilisation allows the aequilibration of intracellular compartment and respirometry buffer (MIR06). Fresh placenta biopsies were chemically permeabilised in ice-cold BIOPS supplemented with saponin 50 mg/ml and incubated in continue shaking for 30 min in cold room. The samples were then washed twice for 10 min in ice-cold BIOPS prior to weighing. Wet weighing of fresh placentas was carried out after permeabilisation thus reducing osmotic variation in water contents. Cryopreserved and snap frozen placenta samples, once thawed, are already permeabilised and ready for weighing and HRR (Kuznetsov et al. 2003; Mardones and González 2003; Giovarelli et al. 2021). For weighing, placenta fragments were placed for 90 s onto blotting paper to wipe off any liquid. Immediately after the recording of the weight, samples were transferred in ice-cold MIR06 buffer prior respirometry analysis.

### High-resolution respirometry (HRR)

Oxygen Consumption Rates (OCRs) of placental specimens (~ 20 mg wet weight) were measured into the 2 ml O2K oxygraph chambers (Instruments Oroboros, Innsbruck, Austria) at 37 °C in stirring MIR06 buffer (Holland et al. 2017). The electrodes were calibrated in MIR06 respiration medium, with a calculated saturated oxygen concentration of 180 μM and the HRR were performed at high oxygen concentration (400 μM) by the addition of hydrogen peroxide. OCRs were expressed in pmol O_2_/min ml to measure the steady-state oxygen fluxes (respiratory rates). We used a specific Substrate-Uncoupler-Inhibitor-Titration (SUIT) protocol 11 with some modifications. Pyruvate (5 mM), glutamate (10 mM), and malate (2 mM) were added to determine complex I (CI) mediated LEAK respiration (state 2 respiration). Then, the complex I oxidative phosphorylation (CI OXPHOS) capacity through CI (state 3 respiration) was stimulated by the addition of ADP (2.5 mM). The addition of cyt c (10 µM) was done to test the integrity of the outer mitochondrial membrane. Subsequent titration with succinate (10 mM) was used to evaluate the maximal OXPHOS capacity through complexes I and II (CI + II OXPHOS). The maximal capacity of the Electron Transfer System (ETS) was evaluated by a 0.5 μM steps titration of the uncoupler protonophore carbonyl cyanide p-trifluoro- methoxyphenyl hydrazone (FCCP). Uncoupled complex II-linked respiration was achieved by the addition of rotenone (0.5 µM) (ETS CII). The respiratory chain was inhibited with the complex III inhibitor antimycin A (2.5 µM) to obtain the non-mitochondrial Residual OXygen consumption flux (ROX). Complex IV (CIV) activity was stimulated by using N,N,N0,N0-Tetramethyl-p-phenyl-enediamine dihydrochloride (TMPD) (0.5 μM) and ascorbate (2 mM), recorded for 5 min and hence stopped with the addition of CIV inhibitor sodium azide (100 mM) to calculate the TMPD autoxidation oxygen consumption. Oxygen fluxes were corrected by subtracting ROX from each steady-state. The Flux Control Ratio (FCR) represents the ratio of oxygen flux in different respiratory states (ROX subtracted) calculated as a proportion of the ETS maximum capacity. Net OXPHOS capacity (P-L) and net ET capacity (E-L) indicate the respiratory capacity available for coupling respiration and ion transport and ADP phosphorylation respectively. The DatLab7 software (Oroboros, Instruments Oroboros, Innsbruck, Austria) was used for data acquisition and analysis. For each fresh placental biopsy, two experimental replicates were performed in parallel.

### Protein isolation and western blot

Placental bioptic specimens (~ 50 mg) were minced and then homogenized through Ultra-Turrax (Ika-lab, Staufen, Germany) in lysis buffer containing 20 mM Tris–HCl (pH 7.4), 10 mM EGTA, 150 mM NaCl, 1% Triton X-100, 10% glycerol, SDS 1% supplemented with a cocktail of protease and phosphatase inhibitors (cOmplete and PhosSTOP; Roche Applied Science, Mannheim, Germany) and incubated 30 min on ice. After centrifugation at 18,000 g for 15 min, proteins were quantified by Bio-Rad protein assay (Bio-Rad, Hercules, CA, USA). 50 μg of total protein were loaded on 4–20% polyacrylamide precast gels (Criterion TGX Stain-free precast gels; Bio-Rad) Before transfer, short photoactivation with UV light made protein fluorescent allowing their immediate visualisation, then the gels were transferred onto a nitrocellulose membrane using a Trans-Blot Turbo System and Transfer pack (Bio-Rad). The membranes were probed using the following primary antibodies: Total OxPhos Rodent WB Antibody Cocktail, mouse (45-8099, ThermoFisher Scientific, Walthan, MA, USA) 1:1000; α vinculin, mouse (V 4505, Merck, Garmstadt, Germany) 1:5000; α Cleaved Caspase-3 (Asp175), rabbit (#9664, Cell Signaling Technology, Danvers, MA, USA) 1:1000; α Cleaved Caspase-9, (Asp315) (D8I9E), rabbit (#20750, Cell Signaling Technology) 1:1000; α Bax, rabbit (#2772, Cell Signaling Technology) 1:1000. The bands were visualized using horseradish peroxidase-conjugated secondary antibodies (Bio-Rad) and the Clarity Western ECL Substrate with ChemiDocMP Imaging System (Bio-Rad). Results were analysed using the Image Lab software (Bio-Rad).

### Histology and imaging

For mitochondrial staining, placental specimens were incubated with MitoTracker Orange CMTMR (M7510, ThermoFisher Scientific) 100 nM in BIOPS for 3 h at 37 °C. MitoTracker marks living mitochondria depending on their membrane potential. After MitoTracker staining, placental specimens were fixed with 4% paraformaldehyde for 1 h and then included in optimal cutting temperature compound (OCT, Bio Optica, Milan, Italy). 10 μm-thick cryosections of placental biopsies were used for both morphological and immunofluorescence analyses. Sections were post-fixed with 4% paraformaldehyde for 10 min, blocked for 1 h with 5% goat serum 0.1% triton-PBS. MitoTracker was visualized by 552 nm laser. Actin cytoskeleton was stained using Alexa 488 fluorescent phalloidin (ThermoFisher Scientific). Nuclei were counterstained with DAPI (1:1000 for 10 min). For immunofluorescence analysis, placenta sections were incubated with specific primary antibodies diluted in blocking solution overnight at 4 °C. The primary antibodies used for immunofluorescences are the following: α Cytochrome c, mouse (556432, BD Bioscience, Franklin Lakes, NJ, USA); α TIM23, mouse (611222, BD Bioscience); α Cleaved Caspase-3 (Asp175), rabbit (#9664, Cell Signaling Technology). After incubation with the appropriate fluorescent-labeled secondary antibodies and DAPI, all the slides were mounted with Fluoroshield mounting medium (Merck). Images are acquired using a Leica TCS SP8 AOBS microscope system using 40X/1.30 oil immersion objective (Leica Microsystems, Wetzlar, Germany).

### Statistics

Grouped analyses of mitochondrial OCR states (Fresh placentas n = 10; Cryopreserved placentas n = 5; Snap Frozen Placentas n = 5) were done by two-way ANOVA followed by post hoc Tukey’s multiple comparisons test. For State 3 cyt c test analysis and MitoTracker intensity quantifications, ordinary one-way ANOVA test with Tukey’s multiple comparisons test has been used. The GraphPad Prism software package (Graph Software) was used. The results are expressed as means ± SEM of the indicated n values. A P value < 0.05 was considered significant. * versus Fresh Saponin Placental specimens [** p < 0.01, *** p < 0.001, **** p < 0.0001]; + versus Cryopreserved Placental specimens [+ p < 0.05, + + p < 0.01, + + + + p < 0.0001]; ^§^ versus Snap Frozen Placental specimens [^§^p < 0.05, ^§§^p < 0.01, ^§§§§^p < 0.0001].

## Results and discussion

**Fig. 1:**
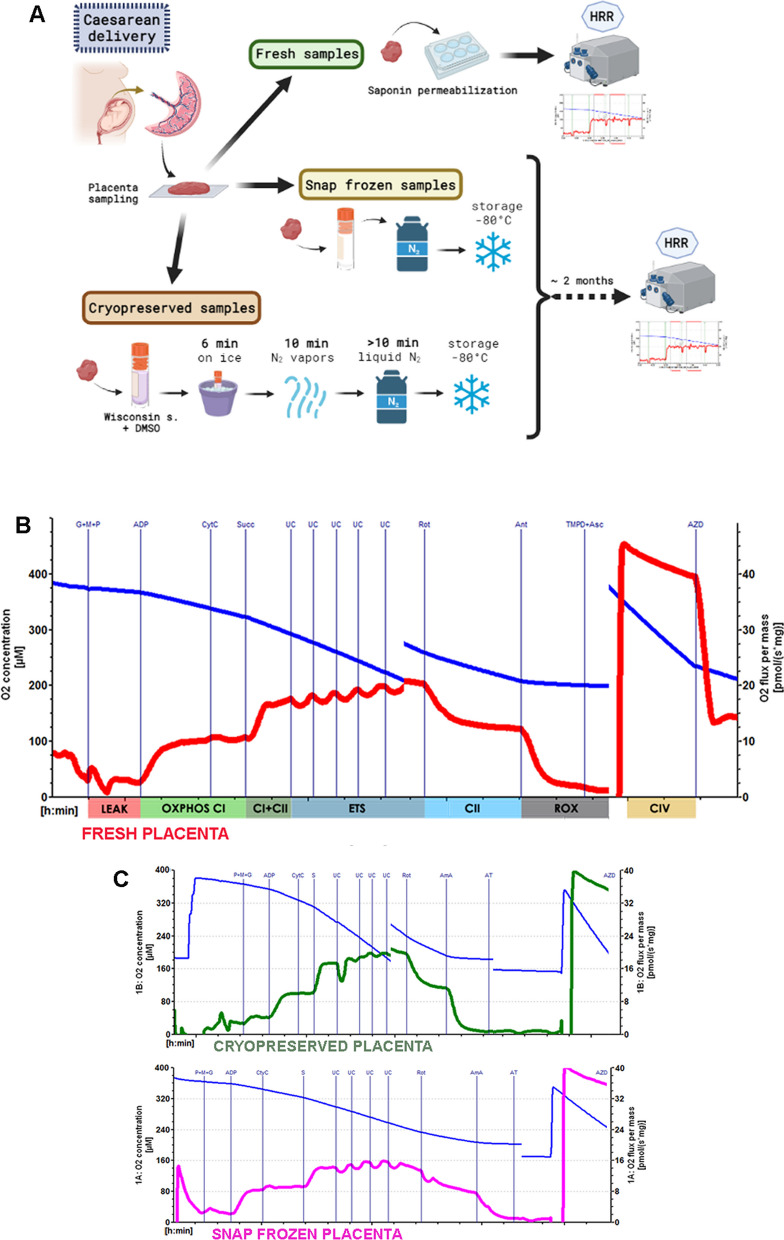
Placental biopsies preparations and respirometry protocol SUIT-11. **A** Placenta biopsies’ preparations flow chart. Fresh preparation, cryopreservation, and snap freezing procedures are represented as shown in materials and methods. Created in BioRender.com. **B** Representative HRR trace of fresh placental biopsy. Blue line: oxygen concentration (µM); red line: oxygen flux [pmol/(s*mg)]. Vertical lines indicate the addition of the indicated chemicals (G + M + P: glutamate, malate, pyruvate; CytC: cytochrome c; Succ: succinate; UC: uncoupler; Rot: rotenone; TMPD + Asc: N,N,N0,N0-Tetramethyl-p-phenyl-enediamine dihydrochloride, ascorbate; AZD: sodium azide). Respiratory states are indicated (LEAK: leak state; OXPHOS CI: complex I-dependent oxidative phosphorylation capacity; CI + CII: complex I + II-dependent oxidative phosphorylation capacity; ETS: maximum electron transfer system capacity; CII: uncoupled complex II-dependent respiratory state; ROX: resting oxygen consumption; CIV: complex IV activity assay). **C** Representative HRR traces of cryopreserved (upper panel, oxygen flux in green) and snap frozen (lower panel, oxygen flux in fuchsia) placenta biopsies

We carried out kinetically controlled HRR measurements of fresh, cryopreserved, and snap frozen human placental chorionic villi specimens applying a specific Substrate-Uncoupler-Inhibitor-Titration (SUIT) protocol. Caesarean placentas were sampled in a central site of the maternal placental side and immediately stored in ice and delivered to research lab. We both analyzed a set of fresh term placentas by HRR and in parallel stored the same by either cryopreservation protocol or a direct snap freezing for later respirometric analyses (Fig. 1A). Our cryopreservation protocol relies on a specific DMSO-based buffer [UW, able to both prolong organ preservation and to prevent cell and organelles swelling (Sakata et al. 1997)] and on a gradual freezing procedure from the on-ice to liquid nitrogen and − 80 °C storage.

**Fig. 2:**
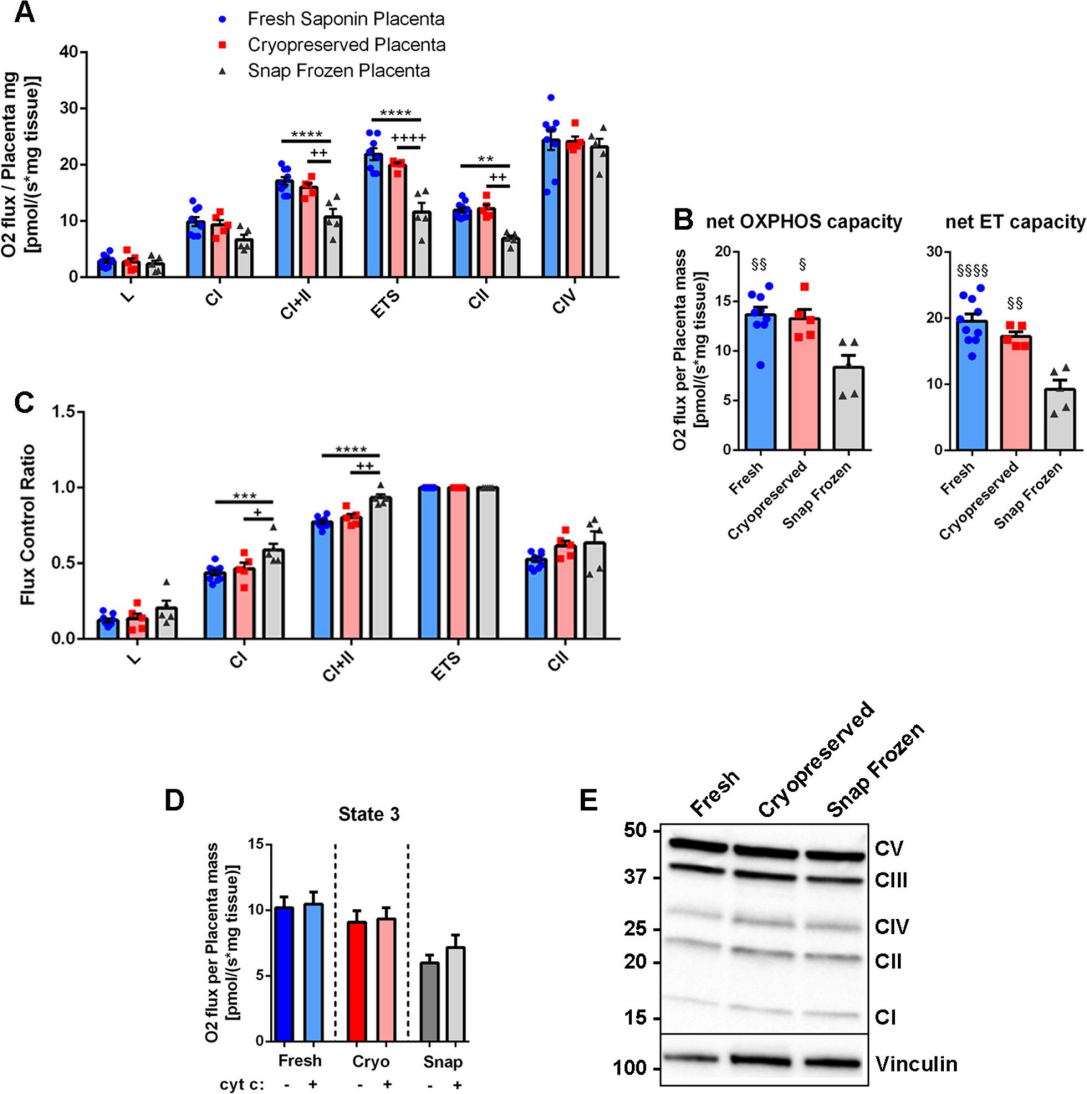
OCRs and respirometric parameters of fresh, cryopreserved, and snap frozen placental biopsies. **A** Oxygen flux values (pmol/(s*mg tissue) of Fresh placentas (n = 10), Cryopreserved placentas (n = 5) and Snap Frozen Placentas (n = 5) in the indicated respiratory states. **B** Net OXPHOS (left panel) and ET (right panel) capacities calculated as P-L and E-L respectively. **C** FCRs of respiratory states as a proportion of ETS state. **D** State 3 respiratory states of Fresh, cryopreserved (Cryo), and snap frozen (Snap) placentas before and after cyt c addition revealing mitochondrial integrity. **E** Representative immunoblotting analysis of OXPHOS complexes and vinculin as loading control in fresh, cryopreserved, and snap frozen placental protein extracts. * versus fresh saponin placental specimens [**p < 0.01, ***p < 0.001, ****p < 0.0001]; + versus cryopreserved placental specimens [+ p < 0.05, + + p < 0.01, + + + + p < 0.0001]; § versus snap frozen placental specimens [^§^p < 0.05, ^§§^p < 0.01, ^§§§§^p < 0.0001]. Values are expressed as mean ± SEM

SUIT protocols enable the study of different respiratory states by oxygen consumption rates (OCRs) measurement through the sequential addition of specific chemicals. The SUIT-11 protocol (https://wiki.oroboros.at/index.php/SUIT-011) was already used for placental specimens and our HRR traces using fresh permeabilised placental biopsies showed oxygen fluxes aligned with already published values of fresh term placentas’ OCRs for all respiratory states (Fig. 1B, values shown in Table 1; Holland et al. 2017, 2018). Cryopreserved biopsies, thawed after 2–4 months of storage, yielded OCRs comparable to fresh samples, demonstrating to retain an intact mitochondrial function and to be effectively permeabilised through the cryopreservation procedure (Mardones and González 2003) (Figs. 1C and 2A). Snap freezing procedure is deemed to uncouple mitochondrial respiration damaging mitochondrial membranes (Larsen et al. 2012); nevertheless, snap frozen placentas, once thawed, do retain a quote of mitochondrial activity (likely taking advantage of UW storage protection). However, they showed a severe reduction of OCRs ranging from ~ 30% in OXPHOS CI and CI + II states, to ~ 50% in ETS maximal capacity and CII respirations compared to fresh and cryopreserved specimens (Fig. 1C and 2A). In particular, snap freezing impaired coupled respiratory capacity leading to the reduction of the net OXPHOS capacity available for phosphorylation of ADP to ATP (Fig. 2B left panel). Moreover, OCR values of OXPHOS CI + CII in snap frozen specimens were close to maximum ETS capacity, indicating a lower ETS spare capacity (Fig. 2A); accordingly, the net ET capacity was reduced (Fig. 2B right panel). Of notice, complex IV (CIV) activity was unchanged across all groups probably due to the nature of the chemical CIV stimulation assay that depends on neither OXPHOS coupling nor mitochondrial membrane integrity. Overall, fresh placental biopsies’ OCRs displayed wider mean standard errors; this could had been caused by the permeabilisation step of fresh specimens as it constitutes a potential technical variability factor. Altogether, these data highlight the cryopreservation protocol effectiveness in maintaining mitochondrial activity also confirming that snap frozen specimens retain a partial ETS activity when maintained stored in an appropriate preservative buffer (Colleoni et al. 2012).

**Table 1:** Placental respiratory parameters

Storage conditions	LEAK state respiration	OXPHOS CI dependent respiration	OXPHOS CI + II dependent respiration	ETS respiration	Uucoupled CII respiration	CIV activity
Fresh	2.90 ± 0.36	10.47 ± 0.91	17.14 ± 0.71	21.89 ± 1.06	11.94 ± 0.46	24.41 ± 1.76
Cryopreserved	2.73 ± 0.76	9.34 ± 0.78	16.00 ± 0.62	19.95 ± 0.41	12.19 ± 0.70	24.16 ± 0.85
Snap frozen	2.39 ± 0.89	6.69 ± 0.87	10.74 ± 1.39	11.61 ± 1.63	6.91 ± 0.51	23.19 ± 1.44

Mean ± standard error. Respiratory parameters are expressed as pmol O2 min − 1 mg − 1

Fresh placentae n = 10; Cryopreserved placentae n = 5; Snap Frozen Placentae n = 5

In line with OCRs values, the flux control ratios (FCRs), that are independent of mitochondrial content and provide an unbiased coupling and ETS gauge, were also consistently aligned in fresh and cryopreserved biopsies (Fig. 2C). By contrast, snap frozen placentas showed higher CI and CI + CII FCR values, thus indicating a reduced maximal ETS capacity (Fig. 2C). Cyt c addition after ADP titration provides a mitochondrial structure quality control able to detect either samples damages or an harsh permeabilisation procedure (Larsen et al. 2012). In both saponin-permeabilized fresh and cryopreserved placentas, state 3 respiration triggered by ADP was not affected by cyt c addition, thus indicating a suitable mitochondrial membrane permeability in both conditions (Fig. 2D). In snap frozen samples, cyt c titration induced a slight increase of respiratory rate, suggesting a partial diffusion of cyt c due to membrane damage (Fig. 2D). Furthermore, since mitochondrial content may affect HRR output, we quantified OXPHOS complexes in placental preparations by western blot detecting no relevant differences after the several storage methods (Fig. 2E).

**Fig. 3:**
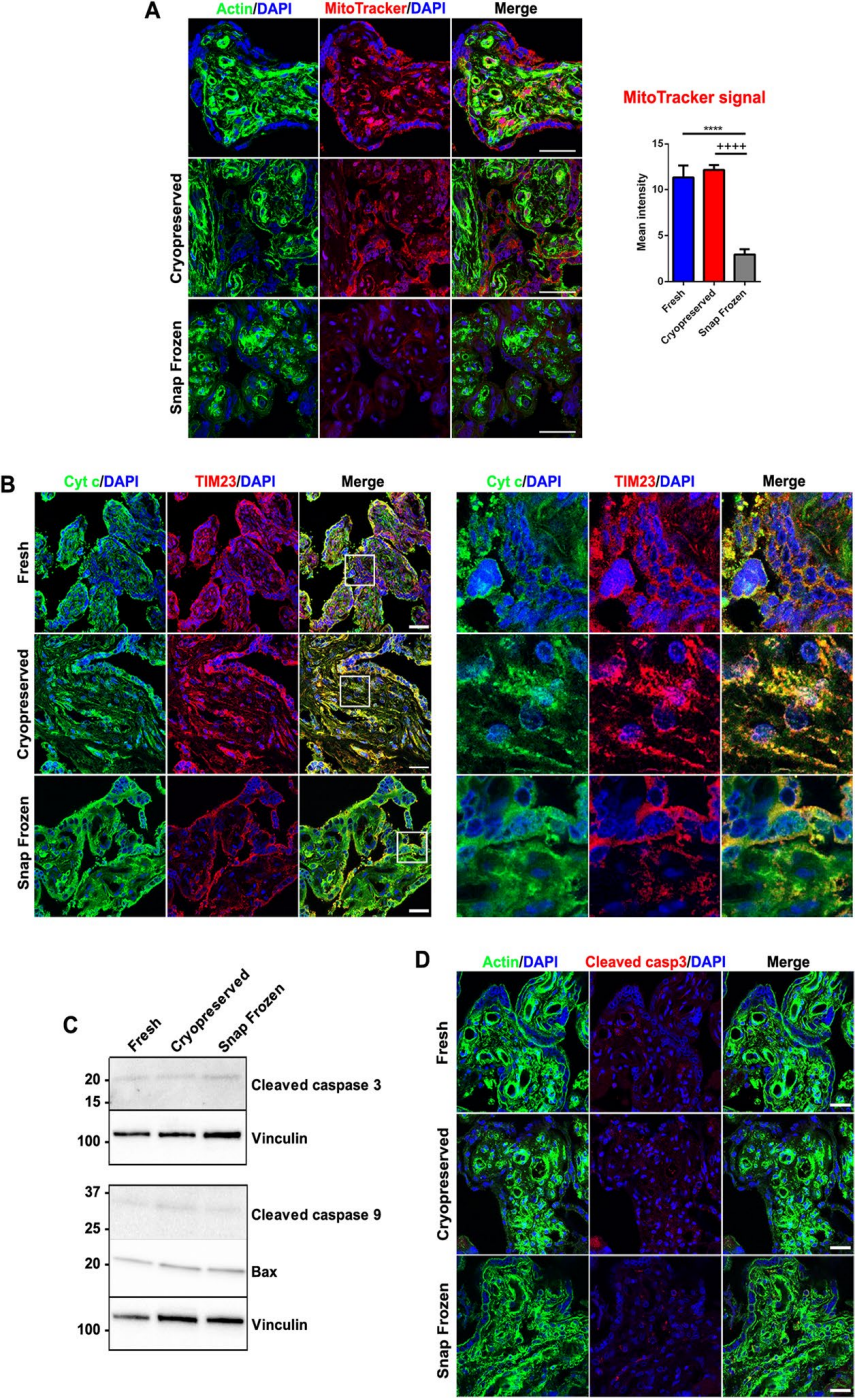
Mitochondrial membrane potential and apoptotic activation after placental biopsies cryopreservation and snap freezing. **A** Confocal fluorescence images of placental sections stained with actin (green), MitoTracker (red), and DAPI (blue). Mitochondrial membrane potential quantifications in fresh, cryopreserved, and snap frozen conditions are provided (right graph). Scale bar: 50 μm. *versus fresh saponin placental specimens [****p < 0.0001]; + versus cryopreserved placental specimens [+ + + + p < 0.0001]. Values are expressed as mean ± SEM. **B** Left panel images: confocal immunofluorescences of placental sections stained for cyt c (green), TIM23 (red), and DAPI (blue). Scale bar: 50 μm. Right panel images: magnifications of left panel’s inserts showing cyt c/TIM23 colocalization. **C** Representative immunoblotting analysis of cleaved caspase 3 (upper panel), cleaved caspase 9, Bax (lower panel), and vinculin as loading control in fresh, cryopreserved, and snap frozen placental protein extracts. **D** Confocal immunofluorescences of placental sections stained for actin (green), cleaved caspase 3 (red), and DAPI (blue). Scale bar: 50 μm

In accordance with the data from HRR measurements, both morphology and membrane potential of mitochondria in cytotrophoblast and syncytiotrophoblast cells were unaffected after cryopreservation, as demonstrated by MitoTracker staining of placental villi (Fig. 3A). As expected, snap freezing procedure reduced the mitochondrial membrane potential (Fig. 3A), though only mildly affecting the overall villous mitochondrial network morphology (Fig. 3B). Moreover, as indicated by cyt c test in HRR (Fig. 2D), snap freezing induced a partial release of cyt c from the cells, resulting in its lower colocalisation with the mitochondrial marker TIM23 (Fig. 3B). Conversely, the consistent cyt c/TIM23 overlapping signals in cryopreserved placentas indicated a conserved mitochondria architecture (Fig. 3B).

To rule out the activation of apoptosis, we analysed key effectors of the apoptotic cascade in cryopreserved and snap frozen specimens thawed and kept at room temperature for ~ 1 h (approximately the time frame of the HRR run). As shown in Fig. 3C and D, the initiator cleaved caspase 9, the executioner cleaved caspase 3, and the pro-apoptotic factor Bax were barely detected by western blot and immunofluorescence. Of relevance, they showed comparable expressions across fresh, cryopreserved, and snap frozen placental biopsies. In this regard, the fast snap freezing procedure, although mechanically triggering a slight cyt c release (Figs. 2D and 3B), was not temporally capable to activate caspase cascade within HRR experimental timing (Fig. 3C, D).

Overall, our results validate the cryopreservation method that enables a convenient and easy storage of placental specimens at the point of delivery. In addition, this comparative study underlines how the common snap freezing methods could sub-optimally affect the storage reliability and the mitochondrial ETS measurement of bioptic samples, potentially masking and confounding clinical data.

## Conclusions

HRR measurements through the new generation of Clark-type electrode devices represent a significant, yet poorly exploited, source of clinical information for both mitochondrial diseases and metabolic conditions related to pregnancy. Therefore, there is a strong need of standardised setting and inter-laboratory protocols’ harmonization able to consistently compare bioenergetic measurements of bioptic specimens and results from different research centres. Here we designed a cryopreservation method, validated it, and showed its reliability when applied to placental biopsies. This method enables a convenient and easy storage of placental specimens at the point of delivery, allowing samples collection from different locations and at different times for subsequent mitochondrial respiratory analyses.

## Data Availability

Further information and requests for resources and reagents should be directed to and will be fulfilled by the lead contact, Matteo Giovarelli (matteo.giovarelli@unimi.it).

## Abbreviations

HRR: High-resolution respirometry
ETS: Electron transport chain
OCR: Oxygen consumption rate
OXPHOS: ADP phosphorylation and metabolic substrate oxidation coupling
BMI: Body mass index
eCS: Elective Caesarean section
UW: University of Wisconsin solution
BSA: Bovine serum albumin
DMSO: Dimethyl sulfoxide
min: Minutes
FCCP: Carbonyl cyanide p-trifluoro- methoxyphenyl hydrazone
ROX: Residual oxygen consumption
TMPD: N,N,N0,N0-Tetramethyl-p-phenyl-enediamine dihydrochloride
FCR: Flux control ratio
OCT: Optimal cutting temperature compound
SUIT: Substrate-Uncoupler-Inhibitor-Titration
CI: Complex I
CI + II: Complexes I and II
CIV: Complex IV
Cty c: Cytochrome c
